# Metabolic enzymes in glial cells of the honeybee brain and their associations with aging, starvation and food response

**DOI:** 10.1371/journal.pone.0198322

**Published:** 2018-06-21

**Authors:** Ashish K. Shah, Claus D. Kreibich, Gro V. Amdam, Daniel Münch

**Affiliations:** 1 Faculty of Chemistry, Biotechnology and Food Science, Norwegian University of Life Sciences, Aas, Norway; 2 Faculty of Ecology and Natural Resource Management, Norwegian University of Life Sciences, Aas, Norway; 3 School of Life Sciences, Arizona State University, Tempe, Arizona, United States of America; University of Arizona, UNITED STATES

## Abstract

The honey bee has been extensively studied as a model for neuronal circuit and memory function and more recently has emerged as an unconventional model in biogerontology. Yet, the detailed knowledge of neuronal processing in the honey bee brain contrasts with the very sparse information available on glial cells. In other systems glial cells are involved in nutritional homeostasis, detoxification, and aging. These glial functions have been linked to metabolic enzymes, such as glutamine synthetase and glycogen phosphorylase. As a step in identifying functional roles and potential differences among honey bee glial types, we examined the spatial distribution of these enzymes and asked if enzyme abundance is associated with aging and other processes essential for survival. Using immunohistochemistry and confocal laser microscopy we demonstrate that glutamine synthetase and glycogen phosphorylase are abundant in glia but appear to co-localize with different glial sub-types. The overall spatial distribution of both enzymes was not homogenous and differed markedly between different neuropiles and also within each neuropil. Using semi-quantitative Western blotting we found that rapid aging, typically observed in shortest-lived worker bees (foragers), was associated with declining enzyme levels. Further, we found enzyme abundance changes after severe starvation stress, and that glutamine synthetase is associated with food response. Together, our data indicate that aging and nutritional physiology in bees are linked to glial specific metabolic enzymes. Enzyme specific localization patterns suggest a functional differentiation among identified glial types.

## Introduction

Glial cells are key to healthy behavioral function and maintain the homeostasis of neurotransmitters and metabolites in the brain. Yet, very little is known about the distribution and functional diversity of glia in the honey bee brain, a system with a decades-long tradition as a model in neurobiology [1,2], and a more recent research focus on highly flexible aging patterns [3,4].

Glial cells play essential roles in neuronal development. They regulate synaptic plasticity, provide neurons with trophic support, and constitute the brain’s primary immune system [5,6]. Glial cells may not outnumber the neuronal population by far, as often asserted, but still represent a major cell population in the brain [7–9]. The various roles of glial cells are reflected in a diversity of glial classes and subtypes. In insects, the identification of glial types is often based on location cues and cellular morphology; more recent approaches also include glial type specific protein expression to identify possible functional differences ([10–15], for the honey bee, e.g., [16,17]). We here adopt a classification system that distinguishes between three classes and five subtypes of glial cells [10,12]. These are, firstly, ‘surface glia’ with two subtypes that reside at the brain’s periphery and form the blood-brain-barrier. A second class of glia is spatially associated with synaptic neuropiles (‘neuropil glia’) and is further subdivided into astrocyte-like and ensheathing glia [11,12,18]. ‘Cortex glia’, the third class, has cell bodies within the soma cortex and forms dense meshworks that envelop neuronal cell bodies [10].

In vertebrate brains some metabolic enzymes appear to be exclusive to glial cells. For example, the presence of glutamine synthetase or of elements of the glycogen breakdown pathway is specific for certain glial subclasses. The first, glutamine synthetase (GS), catalyzes the conversion of glutamate and ammonia to glutamine [19]. Accordingly, the GS mediated glutamate breakdown can remove excess neurotransmitter and can counteract the buildup of toxic ammonia levels [18,20]. High levels of GS have been shown for astrocytes in mammals [18,21], for astrocyte-like glia in *Drosophila* [22], and locusts [11]. Another glial-specific enzyme, glygogen phosphorylase (GP), catalyzes the rate-limiting step in the breakdown of glycogen. While glycogen has been often portrayed as an emergency fuel during hypoglycemia in mammals [23], glycogen degradation by GP may also support neurotransmission under normal conditions [24]. Similar to glutamine synthetase, glycogen phosphorylase in the mammalian brain seems to be almost exclusive to astrocyte glia [25]. However, new reports indicate that neurons, to some extent, can show glycogen metabolism as well [26]. Specific metabolic interactions between glial cells and neurons in the honey bee’s central brain are poorly understood but have been studied in greater detail for peripheral neurons (photoreceptors) and retinal glial cells [27].

As one of the principal models in invertebrate brain research, the honey bee features a complex social behavior, a well-studied neuroanatomy, and relatively simple test paradigms to study learning and memory are established [2]. Adapting such learning protocols [28], research in the last decade has revealed that extreme lifespan and aging differences depend on worker type (‘sub-castes’, [29]), rather than on chronological age [30,31]. For example, short-lived foragers can develop typical aging symptoms within a few days only, while winter bees can exhibit negligible aging for many months [28,32–37]. Symptoms that support worker-type dependent aging include a decline in learning function and stress resilience [32,35,38], as well as rapid mortality dynamics in foragers [36]. Cellular senescence in the brain and other tissues is supported by changed abundance of synaptic and other proteins [33], oxidation of brain proteins [39], by the accumulation of lipofuscin [35,40], by changes in immune function [41,42], and in transcriptional profiles [43]. Brood signals on the colony level [35,44] and the alternative utilization of a yolk protein (vitellogenin) on the molecular level have been identified as key regulators of longevity in worker bees [45–47]. Beyond a particular focus on aging, honey bees are used successfully to study other health related topics, for example the regulation of food intake, effects of nutrients on health, as well as the cross-talk between the brain and the major sites of nutrient utilization and storage [48–50].

A better understanding of glia function and their potential roles in brain function, aging, and nutritional physiology is therefore highly rewarding in the honey bee model. As a first step, we here study cellular localization patterns of the two metabolic enzymes GS and GP within prominent regions of the honey bee’s central brain. Addressing possible roles of GS and GP in aging, starvation, and food response we test for associations of metabolic enzyme levels with these functions.

## Material and methods

### Animals and phenotype identification

Bees (*Apis mellifera carnica* Pollmann) were obtained from apiaries at the Norwegian University of Life Sciences (Aas, Norway). Worker type, chronological age and colony origin were identified for all specimens used in this study. Mature nurse bees, that were between 12–20 days old, were collected from brood combs of colonies housed in our indoor flight-room facilities [35]. To identify their chronological age, these bees had received a paint mark (UniPosca) after hatching in an incubator and then were transferred to experimental colonies from which they were later collected [35]. Foragers were collected from outdoor colonies when returning from foraging flights. To obtain old foragers vs. typically non-senesced, mature controls we used chronological and morphological criteria [51]. In brief, all foragers had a paint mark specifying the days they had spent foraging (“foraging age”), i.e., ≥15 days for the old foragers and ≥5 days for the mature control. In addition, the old group was identified by morphological features that are associated with extended foraging durations [33]. To control for effects of colony origin, specimens for all experiments were collected from at least two colony replicates. In anatomical studies we tested mature nurse bees with an age of 12–20 days. For functional assays and subsequent analyses with Western blotting we either tested the different age groups of foragers or mature nurse bees in the experiments on starvation and food response.

### Dissection and tissue preparation

Specimens were collected into wooden boxes and chilled at 4°C until motionless. After opening the head capsule and careful removal of adjacent, non-neural tissue, e.g., hypopharyngeal glands, brains were transferred into ice-cold fixative (4% paraformaldehyde in phosphate buffered saline, PBS, pH 7.4) for histology or into ice-cold protein extraction buffer for subsequent Western blot analysis (see below).

### Histology and microscopic analyses

After overnight fixation brains were rinsed in PBS. For lipid removal brains were subjected to an ascending and then again descending ethanol series (30, 50, 70, 90, 95, 100% and reverse). After rinsing in PBS, samples were microwaved five times (2 min each) to facilitate antibody penetration. Excessive heating was prevented by submerging the tubes containing the brain samples in a beaker filled with ice water. Tissue samples were then embedded in 5% low melting agarose (Sigma-Aldrich) in PBS, and finally cut to 100μm thick sections using a vibrating blade microtome (Leica VT 1000S, Leica Biosystems).

Sections were rinsed in 0.05% Tween 20 in PBS (PBS-T) and pre-incubated for 1 hr with 2% bovine serum albumin (BSA) in PBS-T. Primary antibody incubation at 1:250 in BSA/PBS-T lasted for 6 days. To prevent tissue degradation 0.01% sodium azide was added to the samples, which were kept at 4°C on a rocking shaker. Subsequent washing with PBS-T was followed by incubation (1 day) with the fluorescence-labeled secondary antibody at 1:500 in PBS-T. Finally tissue sections were washed in PBS-T, dehydrated in an ascending ethanol series and cleared in methyl salicylate.

Images were acquired on a Leica TCS SP5 laser scanning microscope (Leica Microsystems). For multi-channel images the sequential acquisition mode was used to ensure optimal alignment between the different color channels when scanning at high resolution. Lower and higher resolution images were taken with a 10x air, a 20x oil immersion and a 40x oil immersion objective, respectively (numerical apertures = 0.30, 0.75 and 1.25). The according z-step sizes of image stacks were set to 5, 1 and 0.5μm. Image stacks were processed in FIJI/ImageJ, v1.47k (http://fiji.sc, RRID:SCR_002285). Image processing included the application of a Gauss filter with a small kernel size of 1 to attenuate high spatial frequency noise, as well as making 2D projections of image volumes using the maximum projection view mode.

### Antibody characterization and fluorescence markers

Glial cells were identified by using an α-repo serum against a glial-specific transcription factor, which marks the nuclear membrane of almost all glial types in the honey bee (*Drosophila melanogaster* repo produced in *E*. *coli*, rabbit purified serum, RRID:AB_2567900, working dilutions 0.4 μl/ml and 0.2 μl/ml for anatomy and Western blots, gift from J. Urban, Mainz) [52,53]. While the serum has been used previously to identify honey bee glia, its specificity has not been confirmed with Western blotting. Our Western blot tests reveal one band with the predicted size of the putative honey bee repo protein and additional lower weight bands (for more details compare S1 Fig). Glutamine synthetase (GS) was identified with a commercial α-GS antibody (Human glutamine synthetase, amino acid 1–373, mouse monoclonal, BD Biosciences, Cat# 610517 RRID:AB_397879, working dilutions: 0.4 μl/ml and 0.2 μl/ml for anatomy and Western blot). Glycogen phosphorylase (GP) was identified with a commercial α-GP antibody (Human liver glycogen phosphorylase, PrEST, amino acid 299–431, rabbit polyclonal, Sigma-Aldrich, Cat# HPA004119, RRID:AB_1079723, working dilutions: 0.4 μl/ml and 0.2 μl/ml for anatomy and Western blot). The specificity of the α-GS and α-GP antibodies for honey bee brain tissue was confirmed by Western blots, which revealed specific bands within the expected molecular weight range (see Results section). In all anatomical studies we included negative controls, where the primary antibody (α-GS, α-GP) was omitted to assess levels of unspecific staining by the secondary antibody as well as background noise due to autofluorescence. For directly comparing negative controls and the regularly treated samples, all microscopic and image analyses settings were kept constant during scanning of negative controls and the regularly treated test samples (see Results section). All polyclonal, secondary antibodies were purchased from Jackson ImmunoResearch Inc. and were CY3- or CY5-conjugated. To identify the somata of neurons and glial cells we used the nuclear marker DAPI (Sigma-Aldrich).

### Functional assays: Aging, starvation and food response

To test if enzyme levels are associated with aging, we obtained identified phenotypes as described above.

The different starvation protocols available for bees are usually aimed at inducing a uniformly high motivation towards the sugar reward in learning assays. To this end, low stress-related mortality is typically achieved by either restraining movement in holders or by keeping starved bees in small social groups [34,54]. In contrast, we here test effects of severe starvation stress and stress related energy depletion (‘exhaustion’). Therefore, our approach allowed free body and leg movements, while social contact was prevented. The sequence of the protocol was as follows: collected bees were kept overnight in an incubator (32°C) with free access to food (30% sucrose in water) and water. To normalize the satiation state among individuals before treatment, bees were mounted singly in holders, and were force fed with 10μl of 30% sucrose. After releasing, each bee was placed in a separate Eppendorf tube (1.5ml). Food and water were made accessible to bees by inserting a filled pipette tip in a hole pinched in the tube’s lid. In the starved group bees had ad libitum access to water only; the non-starved control in contrast had ad libitum access to a 30% sucrose solution. Mortality was monitored every three hours. After 12hrs, when significant mortality was observed in the starved group (see Results section), all specimens were snap frozen in liquid nitrogen and stored at -80°C for further analyses.

To assess individual differences in food responsiveness we used an established protocol for measuring gustatory responsiveness in bees towards sugary food [28,55]. In brief, collected bees were harnessed in holders, and their antennae were presented with different sucrose solutions in an ascending order from 0, 0.1, 0.3, 1, 3, 10 to 30% sucrose in water. The inter-test interval was longer than 5min to prevent habituation or sensitization effects. Upon stimulation bees were monitored for a positive food response by extending the proboscis (‘tongue’). As a measure of responsiveness, the gustatory response score (GRS) specifies the number of trials a bee would show a positive response. Consequently, a low GRS (1–3) was stipulated for the group of less responsive individuals that only responded to the highest sucrose concentrations. A GRS of 5–7 was stipulated for bees in the highly responsive group.

### Western blot based analysis of protein abundance

Protein extraction and quantitative analyses were performed essentially as we have described previously [39]. In brief, after homogenization in PBS containing 1mM H4EDTA and complete protease inhibitor (Roche Diagnostics GmbH), individual brain samples were centrifuged at 10.000g (10 min, 4°C), and the supernatant was collected. For SDS-polyacrylamide electrophoresis (SDS-PAGE) we used the Mini-Protean Tetra Cell system (Bio-Rad Laboratories) and pre-casted gels (Bio-Rad Laboratories). All gels were loaded with equal sample numbers representing each treatment group and each replicate hive. To minimize confounding technical errors, e.g., edge effects, samples from different treatments were loaded in an alternating lane order, and lane positions were rotated between replicate gels. Following electrophoresis the separated proteins were blotted onto PVDF membranes. Pre-incubation of the membranes with a blocking solution (1% BSA in PBS) was followed by incubation with the respective primary antibody (1:500 for α-GS and α-GP, 1hr). Membranes were then rinsed with PBS-T, incubated with CY5-fluorophore-coupled secondary antibodies (1:500, 1hr, see Histology and microscopic analyses), and finally washed with PBS-T. Membranes with labeled protein bands for GS and GP were imaged using a Typhoon Variable Mode Imager 8600 (Amersham Pharmacia Biotech AB). All blots of a single experiment were scanned simultaneously while taking care to use the full intensity bit-range and avoiding pixel saturation. To assess the total fraction of soluble proteins, we then labeled the membranes with Sypro Ruby Protein Blot Stain (Life Technologies Corp.) and re-scanned them. The Sypro Ruby signal was used as a reference to normalize the GS and GP signals for the total protein amount loaded in each lane. Alleviating some of the challenges posed by selecting a single housekeeper reference [56], the Sypro Ruby signal represents a large fraction of the most abundant proteins. In addition, to rule out that extracts of one individual brain would compromise subsequent protein quantification using the antibodies (α-GS, α-GP) and Sypro Ruby by saturation effects, we have conducted initial Western blot analyses with protein extracts representing different sample concentrations (1/2, 1, 2 brains, see S2 Fig). All grey-scale images were analyzed using the Gel Tool included with ImageJ (see above). To avoid crosstalk between the GS and GP with the Sypro Ruby fluorescence signal, bands corresponding to the molecular weight of the two enzymes were excluded from measurements of the Sypro Ruby reference stain. To test for possible differences in relative protein abundance levels, the GS and GP specific signals were first corrected for the Sypro Ruby protein signal, yielding a relative abundance value. Then each specimen (lane) was assigned a rank ranging between 1 and 9 for each gel with 9 lanes loaded. This allowed including multiple blots in the analyses despite variation in absolute intensity (densitometric) measurements between blots.

### Statistical analysis

Analyses were performed using STATISTICA 11.0 (StatSoft). We used the non-parametric Mann–Whitney U test (MWU) since all intensity data of Western blots had to be transformed into rank data (see previous section). We report Z and P statistics, sample number (N) and degree of freedom (df). Apart from treatment effects, statistics for possible effects of hive origin (biological replicate = colony origin) were calculated for each experiment. Survival statistics for the starvation assay were calculated using Cox’s F survival tests for two-sample groups.

## Results

### Associations of glial cells with glutamine synthetase

#### General distribution

**Fig 1:**
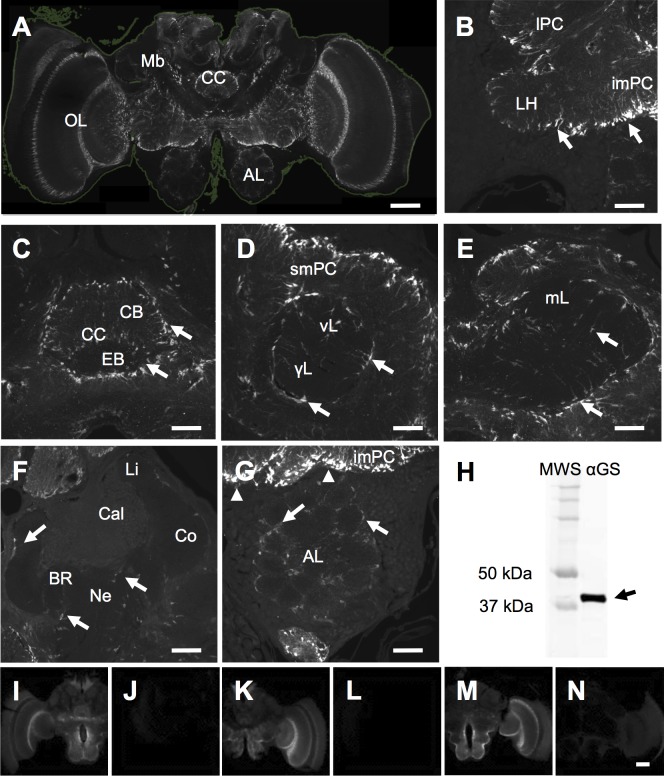
The distribution of glutamine synthetase (GS) in the honey bee brain. (A) Confocal microscopic image stack showing abundant α-GS immunolabeling for different brain areas, including central regions with the mushroom bodies (Mb), central complex (CC), as well as antennal (AL), and optic lobes (OL). The faint green line indicates brain and tissue borders of the brain section. (B-G) Higher resolution images reveal marked α-GS staining (arrows) for the lateral and medial protocerebrum (B; lateral protocerebrum, lPC, inferior medial PC, imPC, lateral horn, LH), for the central complex (C; central complex, CC with central body, CB and ellipsoid body, EB), and for output regions of the mushroom bodies (D, E; vertical lobe, vL and γ lobe, γL, medial lobe, mL). Note that staining was most intense at the periphery of protocerebral neuropiles (B; imPC, lPC, LH; D; superior medial protocerebrum, smPC). In contrast, weaker α-GS immunolabeling was detected in the antennal lobes (G), and only sparse staining in the modules of the mushroom bodies’ calyx regions (F; calyx, Cal, collar, Co, lip, Li, basal ring, BR, neck, Ne). H: Western blots of brain tissue with the α-GS antibody reveal a single band (arrow) close to the expected size of 41kDa (for the complete blot compare S3A Fig). (I-N) With immunohistochemistry we detected a signal only if the α-GS primary antibody was present (I, K, M), whereas controls for unspecific secondary antibody staining and autofluorescence did not reveal marked signals (J, L, N). Scale bar 200μm in A, 50μm in B-G, 200μm in N for I-N.

**Fig 2:**
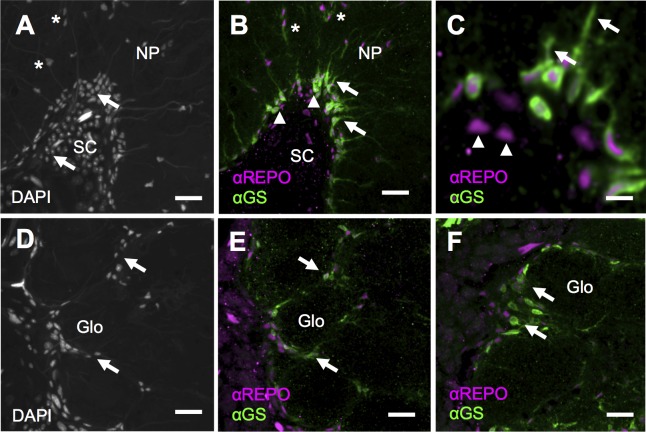
Colocalization of glutamine synthetase (GS) with two types of neuropil glia. (A) Non-glomerular neuropiles (NP), such as proto- and tritocerebral lobes, are surrounded by somacortices (SC). DAPI staining shows the localization of almost all neuronal and glial somata within the soma cortex and the interface to the neuropiles (SC, arrows). Only few cell bodies lie deep in the neuropil (asterisks). (B and C) The glial-specific marker α-repo (magenta) reveals that glial cells are mostly found at the boundary between soma cortex and synaptic neuropil (arrowheads in B). Many of these peripheral glial cells show intense α-GS staining (green). Location and co-localization with α-repo and arborizations that extend into the neuropil (arrows) support that these α-GS positive cells are astrocyte-like glia, a type of neuropil glia. Note that not all α-repo positive glial cells show α-GS staining (arrowheads in C). Yet, α-repo positive glia within the neuropil did show adjacent α-GS, arborization-like staining (asterisks in B). (D) In glomerular neuropiles, such as the antennal lobes (shown), DAPI reveals somata that surround the entire neuropil and also single glomeruli (Glo, arrows). (E and F) α-repo staining (magenta) marks glial cells that outline single glomeruli (arrows), lack arborizations and hence resemble ensheathing, rather than astrocyte glia. Scale bar 20μm in A, B, D, E, 5μm in C, F.

Immunostaining with the anti-glutamine synthetase (α-GS) antibody suggests the presence of GS in different regions of the central brain, yet at very different abundance levels (Fig 1A–1G). Brain regions with intense α-GS immunostaining include the lateral and medial protocerebrum (Fig 1B, 1D and 1G), the central complex (Fig 1C), as well as parts of the mushroom bodies (Fig 1D and 1E). In contrast, staining in the mushroom bodies’ calyx was less intense and sparser (Fig 1F). Likewise, the antennal lobe (arrows in Fig 1G) showed less intense staining, as compared to the medial and lateral protocerebrum (arrow in Fig 1B, arrowheads in Fig 1G). Generally, α-GS positive cell bodies were observed at the outer rim of neuropiles, i.e., the interface, where soma and synaptic neuropil regions meet (arrow in Fig 1B, arrowheads in Figs 1G, 2B and 2C). In addition, α-GS staining was detected within neuropiles, labeling arborizations (Fig 1B and 1D) and few cell bodies (asterisks in Fig 2B). However, α-GS positive arborizations within neuropiles were not identified for the antennal lobes (Fig 1G). Our observations on GS localization are based on the inspection of N = 10 individual brains. Western blots with the α-GS antibody revealed a single band close to the predicted size of a subunit belonging to one of two GS isoforms (41 kDa, Fig 1H), which is in line with data from other insect and mammalian systems [11,19]. For brain sections controls for autofluorescence and unspecific secondary antibody staining were negative (Fig 1J, 1L and 1N), as opposed to test sections that were incubated also with primary α-GS antibody but were otherwise scanned and handled similarly (Fig 1I, 1K and 1M, see Methods section).

#### Associations with glia

We next asked if GS is associated with glial cells, as shown for other systems [11]. To this end we tested for co-localization of the α-GS signal with a glial-specific marker (α-repo [16], Fig 2). A nuclear marker served as a reference for both glial cells and neurons (DAPI). First, we examined possible associations in brain regions that showed a particularly high density of α-GS staining, i.e. the protocerebral lobes (compare Fig 1B and 1G). Comparing the DAPI (Fig 2A) and α-repo signals supports that almost all glial somata (Fig 2B, magenta) outlined the soma cortex (SC in Fig 1A and 1B). We found that α-GS staining appeared to be limited to these peripheral α-repo positive glial somata (Fig 2B and 2C), in contrast to neuronal cell bodies (compare Fig 2A). However, not all α-repo positive glial cells were also α-GS positive (arrowheads in Fig 2C). This suggests that GS is only present in a subset of glial cells (Fig 2B and 2C). Similar to before (Fig 1G), neuropilar arborizations were not detectable for α-GS positive glial cells in the antennal lobes.

We then examined, if GS abundance might differ between different glial subtypes. Among different classification systems for insect brain glia, we base our identification on more recent studies in *Drosophila*, which distinguished between three classes: surface, neuropil and cortex glia [10,12]. We did not observe α-GS staining for the two surface glial subtypes, i.e. peri- and subperineural glia (e.g., compare Fig 1F and 1G). The two neuropil glial subtypes are astrocytes and ensheathing glia. Typically, astrocyte-like glial cells lie just outside of the neuropil and inside the clusters of neuronal somata from where they send extensive arborizations into the neuropil. In accord with these criteria, we found α-GS positive glial cells with somata close to neuropil regions (arrows in Fig 1B, arrowheads in Fig 1G), and long arborizations into the synaptic neuropiles (arrows in Fig 2B and 2C), which suggests the presence of GS in astrocyte-like glia. Yet, such astrocyte like morphology was not found for α-GS positive glia in the antennal lobes. Such ensheathing glia has cell bodies that line the glomerular subcompartments. We reasoned that ensheathing glia would be most reliably identified within the well-structured glomerular organization of the antennal lobes (Fig 2D–2F), in contrast to non-glomerular protocerebral lobes (Fig 2A–2C). However, compared to astrocyte-like glia in the protocerebral lobes, GS-like staining generally appeared weaker in the antennal lobes (compare arrowheads and arrows in Fig 1G). Nevertheless, α-GS staining was clearly detectable around glial nuclei between neighboring glomeruli (arrows in Figs 1G, 2E and 2F). This suggests GS to be present in ensheathing glia. Cortex glia, the third glial class, have cell bodies and arborizations that often are more centrally located within each soma cortex. However, within central brain neuropiles we did not observe spatial distribution patterns for α-GS staining that would support the presence of GS in the cortex glial type.

In all, GS-like immunoreactivity appeared specific only for neuropil glia, i.e., astrocyte-like and ensheathing glia, and the abundance of GS-like immunoreactivity differed considerably between brain regions.

### Associations of glial cells with glutamine phosphorylase

#### General distribution

**Fig 3:**
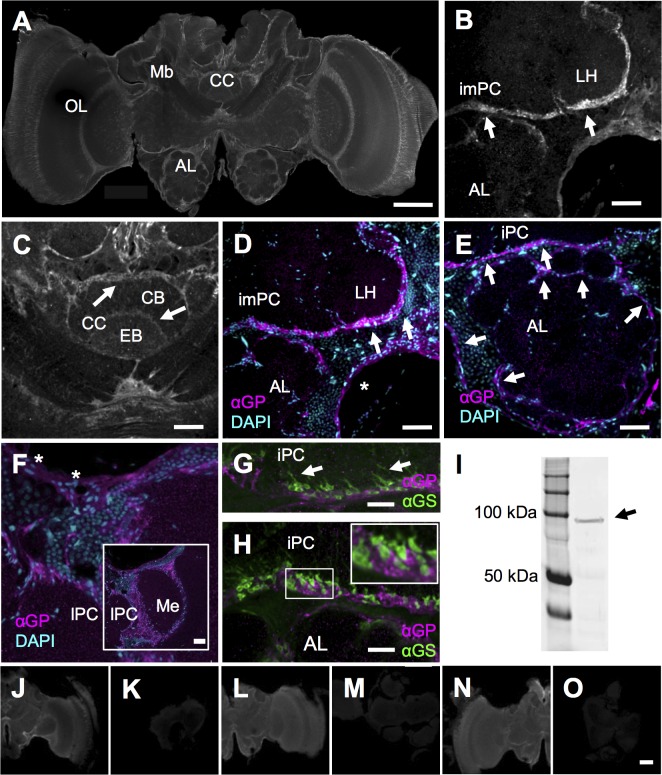
The distribution of glycogen phosphorylase (GP) in the brain. (A) Abundant α-GP immunostaining was detected in different brain areas. The image stack shows α-GP staining in similar brain regions as Fig 1A for α-GS (antennal, AL, central complex, CC, mushroom bodies, Mb, optic lobes, OL). (B and C) Higher resolution images confirming α-GP signals (arrows) for the protocerebral lobes (B, inferior medial protocerebrum, imPC, lateral horn, LH) and the central complex (C; central complex, CC, central body, CB, ellipsoid body, EB). (D-F) Co-localization with the nuclear marker (DAPI, cyan) demonstrates that most intense α-GP signals (magenta, arrows) outline the neuropil of non-glomerular protocerebral lobes (D, lateral horn, LH, inferior medial protocerebrum, imPC) and mark borders of the antennal lobe’s (AL) glomerular neuropiles (E, arrowheads). These localization features suggest that GP is present in ensheathing glia (arrows in D, E, compare A). In addition, α-GP staining at the brain’s periphery suggests GP is present in surface glial subtypes that form the blood brain barrier (asterisks in F). The lower resolution inset in F depicts the location of neuropiles shown in F (lateral protocerebrum, lPC, medulla, Me). (G and H) Co-staining for α-GP and α-GS in cell bodies located between the AL and protocerebral neuropil regions. In contrast to α-GS immunostaining (green), arborizations (arrows) into the neuropil were not evident with α-GP, suggesting different cellular localization for both enzymes. Inset in H with a high-resolution image showing that α-GP and α-GS do not co-localize, suggesting that the enzymes are specific for different glial types. I: Western blots of brain tissue with the α-GP antibody reveal a single band with the expected size of about 97kDa (arrow, for the complete blot compare S3B Fig). (J-O) Using the same microscopy settings, we only detected immunosignals for samples incubated with the α-GP primary antibody (J, L, N) but not in controls for unspecific secondary antibody staining and autofluorescence (K, M, O). Scale bar = 200μm in A, 50μm in B-F, 20μm in G, H, 200μm in O for J-O.

Unlike GS, glutamine phosphorylase (α-GP) immunoreactivity was abundant in all brain regions we inspected (Fig 3). These include lateral and medial protocerebrum (Fig 3B), the central complex (Fig 3C), the mushroom bodies’ output lobes and calyx regions (Fig 3A), as well as the antennal lobes (Fig 3E). The intensity of α-GP immunosignals generally appeared weaker, and less defined as with α-GS. Also, we did not observe marked immunopositive arborizations into neuropiles that we had found with α-GS. Rather, labeling seemed more confined to the interface between somatic and neuropilar areas and therefore may suggest a different cytosolic localization or different glial type specificity than we found for GS (arrows in Fig 3A and 3E, see below). Our observations on GP localization are based on the inspection of N = 9 brains. Western blots reveal a dominant band close to the predicted size of 97 kDa (arrow in Fig 3I). Controls for autofluorescence and unspecific secondary antibody staining were negative (Fig 3K, 3M and 3O), as compared to regularly treated sections that were incubated also with the primary α-GP antibody (Fig 3J, 3L and 3N).

#### Associations with glia and GS

Marked α-GP staining was detected at the periphery but not in more central areas of soma cortices (compare e.g., Fig 3B, 3D and 3F). Again, this distribution pattern is similar to what we showed with α-repo positive glial cells and suggests a glial-specific localization also for GP (compare e.g., Fig 2A and 2B). Co-localization tests with α-GS and α-GP reveal immunostaining for both enzymes within the same areas at the interface between neuropiles and soma cortices (Fig 3G and 3H). Our high-resolution images do not support, however, that α-GP stains the same α-GS positive astrocyte-like glia with projections into the neuropiles (e.g., Fig 3G and 3H). Rather GP appears restricted to the outer edges of neuropiles and, hence, seems most abundant in a type of ensheathing glia (Fig 3F, 3G and 3H, see Discussion). For glomerular neuropiles of the antennal lobe, marked α-GP staining was found to be associated with glial nuclei that surround single glomeruli (Fig 3E), again suggesting the presence of α-GP in ensheathing glia. Finally, marked α-GP immunostaining was detected also at the periphery of the brain where the neurolemma forms the blood brain barrier. Here, surface glia with nuclei, that show a typical elongated shape, were found to be associated with α-GP immunostaining (asterisks in Fig 3F).

Together, our anatomical data suggest that both enzymes, GS and GP are associated with glial cells. The different spatial distribution patterns and distinct morphological features suggest that GP and GS are expressed by different classes and subtypes of glial cells.

### Age-related changes of GS and GP levels within the honey bee brain

To test if GS and GP levels may change during aging, we compared relative protein abundance in brains of old bees with mature, typically non-senesced, controls [51]. As previously, individuals of the old group were forager type bees with a foraging duration of ≥15 days that were additionally identified by age-specific body wear, including worn wings and hairless patches on head and thorax [33]. We assessed brain GS and GP levels with semi-quantitative Western blot analysis using the enzyme specific antibodies (see Figs 1H and 3L).

**Fig 4:**
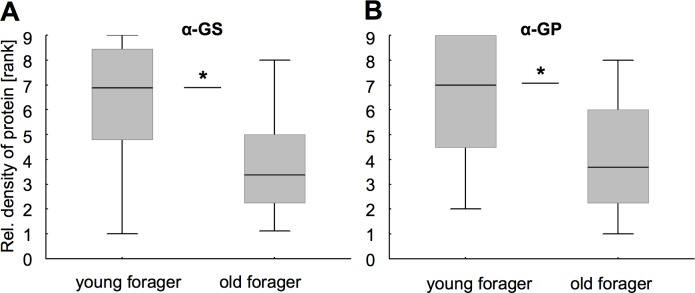
The relative abundance of glutamine synthetase (GS) and glycogen phosphorylase (GP) was reduced in the old group of foragers. (A and B) Relative protein abundance of GS (A) and GP (B) in brains of the old group (foraging duration ≥ 15 days) as compared to the young group of foragers (foraging duration ≥ 5 days). Box plots indicate medians and 25/75 percentiles for rank values assessed by semi-quantitative Western blotting. Ranks were calculated from GS and GP densitometric values, normalized to the total protein staining (Sypro Ruby). Asterisks depict significance (* P<0.05; for detailed statistics see the Results section).

For both enzymes we found that relative GS and GP abundance levels were reduced in the old as compared to the mature control (MWU for GS: df = 1, N_mat/old_ = 12/13, Z = -2.02, P<0.05, Fig 4A; MWU for GP: df = 1, N_mat/old_ = 11/14, Z = -2.03, P<0.05, Fig 4B). This effect was only present, when both foraging duration and morphological criteria of senescence were stipulated to minimize heterogeneity that is typical for the old group (data not shown, [31,33]). We did not observe replicate effects between the different colonies for GS (MWU for GS: df = 1, N_C1/C2_ = 13/12, Z = 0.68, P = 0.4956). For GP, however, a difference between sampled colonies was detected (MWU for GP: df = 1, N_C1/C2_ = 13/12, Z = 2.26, P<0.05).

### Associations between GS and GP levels with severe starvation stress

**Fig 5:**
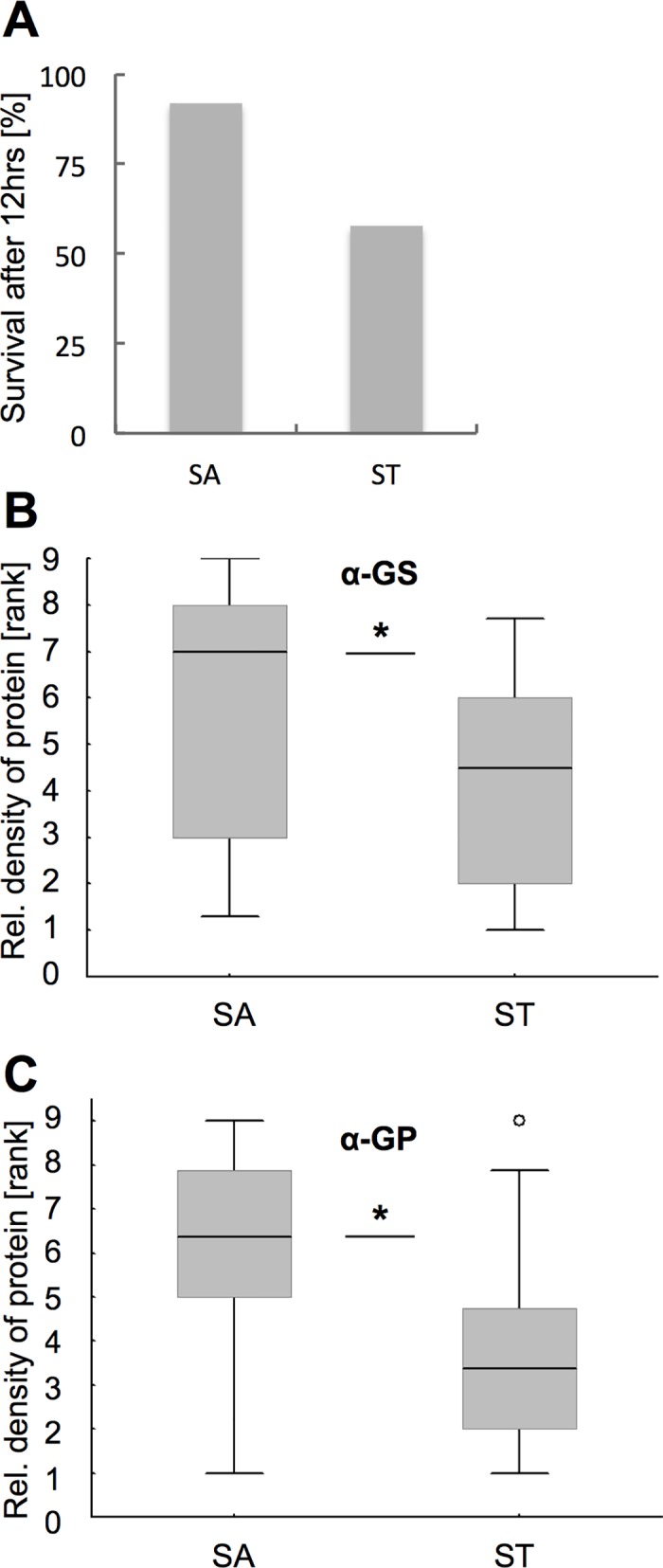
Severe starvation stress reduced the relative abundance of glutamine synthetase (GS) and glycogen phosphorylase (GP). (A) Mortality after 12hrs was significantly higher in the starved group as compared to controls that were allowed to feed ad libitum (detailed statistics in the Results section). (B and C) Relative protein abundance of GS (B) and GP (C) in brains of the starved (ST) and the satiated control group (SA). Box plots indicate medians and 25/75 percentiles for ranked protein abundance values. Asterisks depict significance (* P<0.05; for detailed statistics see the Results section).

To test how metabolic enzyme levels in the bee brain are affected by severe nutritional stress, we subjected nurse bees to a starvation protocol that lasted for 12 hours. As compared to satiated bees, this treatment caused a significantly higher mortality (>40%) in the starved group (Cox’ F, df = 1, N_sat/starv_ = 50/64, F = 6.29, P<0.001, Fig 5A) with a sharp increase in mortality between 9 to 12 hours (data not shown). Our semi-quantitative Western blot analyses show that severe starvation stress was associated with reduced relative abundance of both enzymes, GS (MWU for GS: df = 1, N_sat/starv_ = 17/14, Z = -2.01, P<0.05, Fig 5B) and GP (MWU for GP: df = 1, N_sat/starv_ = 18/16, Z = -2.23, P<0.05, Fig 5C). We did not observe replicate effects, i.e. differences between sample colonies were not significant (MWU for GS: df = 1, N_C1/C2_ = 15/16, Z = -0.12, P = 0.9053; MWU for GP: df = 1, N_C1/C2_ = 17/17, Z = 1.16, P = 0.2476).

### Associations between GS and GP levels with food response

**Fig 6:**
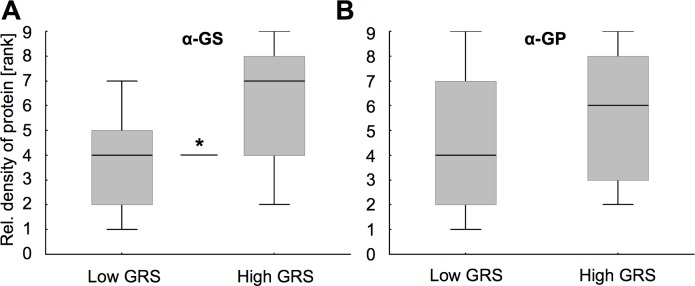
Gustatory responsiveness (GRS) to sucrose was associated with relative abundance of glutamine synthetase (GS) but not with glycogen phosphorylase (GP). (A) Relative GS abundance was higher in brains of bees with high sucrose responsiveness (High GRS), as compared to the group with low responsiveness (low GRS). (B) No such association was evident for GP. Box plots indicate medians and 25/75 percentiles for ranked protein abundance values. Asterisks depict significance (* P<0.05; for detailed statistics see the Results section).

When touching their antennae with different sugar concentrations, bees can extend their proboscis (‘tongue’) allowing measuring their food sensitivity. To test for possible associations with glial-specific metabolic enzymes, we again analyzed relative protein abundance and now contrasted individuals with high and low gustatory response scores (GRS, see Methods section). Low GRS and, thus, a less sensitive food response was associated with a lower relative abundance of GS (MWU: df = 1, N_low/highGRS_ = 13/14, Z = -2.32, P<0.05, Fig 6A). In contrast, we did not find a relative abundance difference for GP between the low and high GRS group (MWU, df = 1, N_low/highGRS_ = 14/13, Z = -1.1466, P = 0.2515, Fig 6B). Again, protein abundance levels were not different between colony replicates (MWU for GS: df = 1, N_C1/C2_ = 13/14, Z = -0.12, P = 0.9029; MWU for GP: df = 1, N_C1/C2_ = 14/13, Z = -1.59, P = 0.1127).

## Discussion

We identified different spatial distribution patterns for glutamine synthetase (GS) and glycogen phosphorylase (GP) in the honey bee brain, suggesting the presence of both enzymes in glial cells. These results support functional differences between glial subtypes and between different brain compartments. Additionally, our quantitative data indicate that survival-critical traits such as aging, starvation stress and food response can be linked to changed levels of GS and GP.

### Spatial and cellular distribution of GS and GP: Subcellular distribution

The highest abundance of GS was present in midbrain neuropiles, in particular around protocerebral lobes and in the central complex (Fig 1B, 1D and 1G). Within the mushroom body, different GS abundance levels appear to reflect the division into midbrain output regions (medial, vertical and γ lobe; Fig 1D and 1E) and the more peripheral input regions (calyx neuropiles; Fig 1F). Similarly, the peripheral antennal lobes showed generally less GS staining (Fig 1G) than midbrain neuropiles. High GS abundance in the central complex and around mushroom body output lobes is in accord with studies in two other insects (*Schistocerca gregaria* [11]; *Drosophila melanogaster* [22]). A lower GS abundance in the calyx, however, may correspond with a markedly fewer glutamate transporters, as shown in *Drosophila* [57]. Because of its role in glutamate metabolism, it can be expected that the distribution of GS resembles that of glutamate. Immunolocalization of glutamate in the honey bee brain generally corroborates this [58]: highly abundant glutamate in lateral and medial protocerebral lobes is contrasted by much lower levels in the calyx region, for example.

Apart from being a central metabolite, the relevance of glutamate for neuronal network plasticity was addressed by a number of studies in bees [59]. Regarding a role in neurotransmission, GS mediated turnover of excess glutamate from the synaptic cleft would suggest spatial associations of GS with neuronal glutamate receptors and with other elements of glutamate neurotransmission. Such associations are supported by similar distribution patterns of GS and the glutamate specific NMDA receptor (AmNR1). Specifically, high NMDA receptor levels in midbrain protocerebral lobes and the central complex again contrast with lower levels in calyx neuropiles [60]. However, the mRNA levels for the NMDA receptor subunit NR1 (*nmdar1*) in the calyx are much higher than what would be expected from (i) the low levels of its protein product (NMDA receptor, [60]), from (ii) the lower levels of glutamate [58], or from (iii) the low GS levels we found for the calyx (Fig 1F). Similarly, mRNA levels of a putative glutamate transporter (AmEAAT) were found to be conspicuously high within the calyx for the mushroom bodies’ Kenyon cells [61]. Possible explanations for the spatial discrepancies of mRNA and protein data include post-translational modifications. Nevertheless, while mRNA and protein distribution data underline the fact that elements of glutamate neurotransmission are present in all brain region, our data on GS supports earlier assumptions on substantial spatial differences for glutamate metabolism within the bee brain [59].

For GP such differences between brain regions were not evident (Fig 3A–3F) and profound spatial differences in glycogen metabolism are, hence, not supported. This seems in line with the more or less homogenous distribution of the substrate of GP, glycogen, as reported for the *Drosophila* brain [62]. Also in contrast to GS, we report that GP staining often appeared more diffuse, with stained puncta within the neuropiles (e.g., in more central regions of the lateral horn, Fig 3D, and the medulla, Fig 3F). While, such small, scattered signals typically indicate staining artifacts, we cannot completely rule out the presence of GP within neuropiles.

Gross spatial distribution patterns of α-GS and α-GP staining (Figs 1A and 3A) are in accord with glial-specific α-repo staining (Fig 2), and with earlier descriptions of glia in the brains of honey bees [16,17], in locusts [9,11] and in *Drosophila* [10,12,13]. Specifically, for both enzymes we found immunopositive labeling at the interface between soma cortices and synaptic neuropiles, hence, in areas that are typically devoid of neuronal somata. Similar to a previous study in the locust’s central complex [11], we found that not all α-repo positive glial cells appear to express α-GS (e.g., compare Fig 2A with 2C). Adding to this, our data indicates that GS is present in both types of neuropil glia, i.e., in astrocyte-like and ensheathing glia but not in surface or cortex glia. In agreement with studies in locusts and *Drosophila* [11,22], GS positive astrocyte-like glia gave rise to extensive arborizations into the neuropiles, with the exception of the antennal lobes’ glomerular neuropiles. Morphologically similar glial structures with extensive branches within the neuropiles may represent a second type of astrocyte-like glia (Fig 2B), which differs from those with peripheral somata typically described for *Drosophila* [12].

Lastly, *Drosophila* has two GS isozymes–a cytosolic and a mitochondrial–with slightly different molecular weights [63]. Yet their abundance in brain tissue is not clear. A nucleotide BLAST search for *A*. *mellifera* also suggests the existence of two GS isoforms in the honey bee. Since our Western blot data on bees revealed a single band only, it is conceivable that only one isozyme is dominant in honey bee brain tissue. Alternatively, the antibody may bind to only one of two isoforms in the honey bee brain. Therefore, we cannot rule out that both, anatomic distribution and enzyme abundance changes are specific for only a subset of glial cells, which contains one of two GS isozymes.

Like GS, GP was found in glia around neuropile and also around glomeruli of the antennal lobes, suggesting the presence in ensheathing glia. In contrast to GS, extensive α-GP-positive arborizations were not evident from our image data (Fig 3G and 3H). Also unlike GS, we did not find conclusive evidence for GP within the large non-glomerular protocerebral lobes (Fig 3B). Hence, GP does not seem associated with astrocyte-like glia, which is further supported by the lack of co-localization between GS and GP in the protocerebral lobes (Fig 3H). This may represent a fundamental difference to vertebrate systems, where glycogen metabolism and highest levels of the brain isoform of GP are commonly associated with astrocytes but not other glial types [24,64]. Based on different localization and morphology, we classify GP positive glia at the boundary between soma cortex and neuropile as a type of non-astrocytic, ensheathing glia. Finally, GP at the surface of the brain (Fig 3F) supports that also surface glia expresses this enzyme. It is conceivable that only one of the two types of surface glia contains GP. Yet, our data show GP around two layers of nuclei, part of them clearly elongated [10] and, thus, resembling perineural and subperineural glia. Nevertheless, because of the more faint staining at the periphery and the close apposition of the two glial layers, we suggest that subtype specific localization is best addressed by future studies using higher resolution imaging, perhaps in cell cultures. Similarly, such studies may address if GS and GP positive ensheathing glia in the antennal lobe of the honey bee can also belong to two subtypes, as it is the case in *Manduca sexta*. That way, GS and GP again may be a key feature that distinguishes astrocyte-like, so-called complex glia and simple glia, which envelops neuropilar compartments [65]. If separate localization of GS and GP also in the antennal lobes holds true, then GS and GP would stain entirely different, non-overlapping glia populations throughout the different brain areas.

Lastly, while co-localization with glial-specific markers (GS) and glial-typical anatomic distribution (GS and GP) suggest an exclusive localization of GS and GP in glia, we cannot entirely rule out that there might be limited co-localization also with other non-glial cells.

### Associations of GS and GP levels with aging and other functions critical for survival

Age and stress-related disruption of metabolic homeostasis mechanisms is regarded as a main route of brain aging, which is why we here focused on potential changes in glial-specific enzyme abundance. We found that relative abundance of GS and GP was reduced in the group of old foragers, as opposed to the mature control. Such age related protein reduction may be attributed to lowered protein expression, changed protein degradation and other factors that affect proteostasis. However, the relative contributions of these factors are difficult to disentangle and can differ among the main aging and disease models [66]. Another factor, cell loss, has been repeatedly shown to accompany aging, however its extent and contribution to normal aging is not yet sufficiently understood ([67], and references therein). Similarly, based on our data we cannot exclude that lower protein levels in aged bees are perhaps partly explained by gial cell loss. Adding to the age affect in foragers, we also report a colony effect for GP. Such colony effects on metabolic enzyme levels may be indicative of differences in colony nutritional status. However, such effect was only revealed by testing GP, not GS, and is also not revealed by testing nurse bees in starvation and food response assays.

Among the behavioral decline patterns in old forager bees, are reduced olfactory and tactile learning as well as changed spatial extinction in home finding tests [31]. In particular, the reduction of GS levels may cause senescence by affecting glutamatergic transmission. However, in contrast to vertebrates, where impaired glutamate recycling causes over-excitation (excitotoxicity)[18], changed glutamate abundance in insects would likely impair the function of inhibitory circuits [68]. In addition, cellular senescence due to reduced glutamate-glutamine conversion can lead to the accumulation of toxic ammonia levels [18], which causes the swelling of astrocytes triggering other mechanisms that further compromise astrocytic homeostasis [69]. Moreover, the role of GS mediated glutamate homeostasis for cognitive dysfunction is supported by reduced GS levels found in Alzheimer’s disease models [70] and by associations with other neurodegenerative pathologies [71]. In contrast, less is known about the role of glycogen phosphorylase in aging. For vertebrates, it seems likely that reduced glycogen phosphorylase reduces an individual’s resilience to metabolic stress. Specifically, in the case of acute hypoglycemia (glucose deficiency) the aged brain would not be able to activate glycogen as an alternative energy resource, which would contribute to increased age related frailty or mortality. For insects the availability of GP is even more critical for survival because of its central role in trehalose synthesis (see below). Perhaps supporting a link between the age-related reduction of GP and lowered metabolic stress resistance are findings demonstrating that over-aged nurse bees [38] and old foragers [72] are less resilient to starvation stress than non-senesced control groups.

To induce severe metabolic stress we starved mature nurse bees for 12hrs, and found that starvation was associated with increased mortality and reduced levels of GS and GP. Hence, our data imply that a reduced availability of these metabolic enzymes is associated with physiological changes that precede stress related death and may reflect ceasing glutamate and glycogen metabolism. One likely explanation for the lowered enzyme abundance is the activation of degradation pathways, such as autophagy, by severe and prolonged starvation stress [73,74]. Interestingly, lowered GS and GP levels appear to contrast with events during early starvation. Such starvation effects on carbohydrate metabolism enzymes are well studied for the insect fat body, which responds to energy shortage by GP activation in order to break down glycogen and produce trehalose. For example, in starving silkworm larvae, the increased GP activity and trehalose production during the first 3–6 hours is followed by a gradual decrease of trehalose in the fat body [75,76]. It remains to be shown how the different mechanisms of metabolic enzyme activity regulation [24,77] during early starvation in the fat body map on those in the bees’ glial cells.

Gustatory responsiveness provides information about the readiness of a bee to feed on sugary food that contains lower versus higher amounts of sucrose. This response was linked to a number of different factors, including pheromones that signal colony-specific environments [78], genetic background [79], foraging preference for sugar or protein rich food [80], and resilience against metabolic stress [72]. Gustatory responsiveness is not fixed however but is strongly up-regulated by acute starvation [81]. Here we show that gustatory responsiveness was associated with relative abundance of one of the two metabolic enzymes: GS. An interaction with starvation is rather unlikely as the satiation state of all bees was normalized by feeding them similar amounts of sugar water before testing. In conclusion, our data for GS adds to recent studies in the bee’s fat body [49] and brain [82], which could link pathways with conserved roles in metabolism and foraging behaviors to gustatory responsiveness.

## Conclusion

Our anatomic data suggest that different types of glia can be distinguished based on GS and GP expression. In addition, the lack of GP in astrocyte-like glia suggests that the cellular localization of the glycogen breakdown might differ from vertebrate astrocytes. We anticipate that GS and GP both provide useful markers for future studies that attempt a more refined functional classification of glial populations in honey bees and other insects. Such reappraisal of previous glial cell classification systems already suggested different subtypes of astrocyte-like glia, for example, and the annotation of additional glial cell classes [15]. Concerning aging, using glial-specific markers in the honey bee now allows to study in more detail the associations of a protein with age protective characteristics (vitellogenin) and glial cells, which we have previously reported [83].

We hope our findings can inspire future studies in the honey bee model to better understand the role of neuron-glia interaction in memory formation, aging, and nutritional physiology.

## Supporting information

S1 Fig Western blots to test the specificity of the *Drosophila* α-repo serum for honeybee brain tissue. The α-repo serum was previously used for anatomic localization of glial cells in different insect species, including the honey bee, and the resulting anatomic data has been shown to conform with alternative glial localization approaches ([11,52,53,84,85]; for an overview on repo as a glial marker target see [86]). To our knowledge, however, no data is published that demonstrates the specificity of the antibody serum with Western blotting.
We found that Western blotting of nuclear extracts from nurse brains revealed multiple bands, all below 50kDa with the largest size band at ca. 45 kDa (arrows, 2 representative blots for N = 10 brain samples). To predict the size of the putative honey bee protein, we performed a BLAST against the *Drosophila* sequence (NCBI BLAST, http://blast.ncbi.nlm.nih.gov/Blast.cgi, RRID:SCR_004870) and found the result to be consistent with the recently annotated gene *retinal homeobox protein Rx2/repo*; (LOC410151, *A*. *mellifera*). With 48.7kDa, the calculated molecular weight of the predicted protein product (*A*. *mellifera* retinal homeobox protein Rx2, XP_016772105) is considerably lower than in *Drosophila* (70kDa, [53]) but approximates the size of the largest size band that we have detected in Western blots (arrows).
However, our Western blots also reveal a number of additional bands below 30kDa, with a prominent band at ca. 18kDa. While we cannot rule out that these may indicate a relatively low specificity of the antibody, we report that all additional bands have a lower molecular weight than the largest size band at ca. 45kDa. This suggests that these bands represent degradation products of the full-size protein. Such degradation may be due to the more extensive lysate treatment that was needed to collect nuclear fractions of brain extracts (below) and, hence, to account for the nucleus-specific localization of the repo protein (compare [53]).
**Nuclear fractionation.** To collect nuclear extracts, we essentially used a hypotonic buffer based protocol. Briefly, two brains were collected into 15μl hypotonic buffer I (20 mM Tris-HCl pH 7, 10 mM NaCl, 3 mM MgCl_2_) to which 0.75 μl 10% NP-40 were added. Brief vortexing was followed by initial centrifugation for 10min at 3000g and 4°C. The resulting pellet containing the nuclear fraction was washed with the hypotonic buffer I, re-suspended in 15μl hypotonic buffer II (5mM EDTA, 1% Tween-20, 0.5% SDS) and briefly vortexed. After a second centrifugation for 10 min 15000 g at 4°C, the supernatant containing the nuclear fraction was collected and further processed as described in the Material and Methods section for GS and GP.
(TIFF)

S2 Fig Titration tests and loading controls. (A, B) Titration tests with antibodies confirm that protein extracts that are equivalent to 0.5 and 1 brain do not saturate the densitometric measurement system (shown here for the α-glutamine synthetase, α-GS, antibody). Prior to semi-quantitative Western blot quantification (compare Figs 4–6) with the two antibodies used, we tested their densitometric response in relation to the amount of brain tissue (‘brain equivalents’) used for gel loading. To this end, 3 independent samples, each with two brains, were pooled (‘stock’) and were then diluted with Laemmli buffer. The resulting protein samples were equivalent to the protein amount of 2 brains (‘stock’), of 1 brain and of ½ brain. (A) Relative protein abundance of GS (in %) shown for three dilutions series, each normalized to the densitometric values measured for the protein ‘stock’ sample, i.e., for the 2-brain equivalent. We found that densitometric values for 1 and 1/2 brain equivalents are smaller than for the 2 brain equivalents. Hence, tissue amounts used to compare different experimental groups (Figs 4–6) will not cause unwanted saturation effects. (B) Representative Western blot showing titration series labeled with the α-GS antibody, and reveal that the GS-band at ca. 41kDa correlates with protein sample concentration. (C) As a reference for normalizing α-GS and α-GP signals to the total fraction of soluble proteins loaded, we re-labeled all blots with Sypro Ruby Protein Blot Stain (Life Technologies Corp.). To avoid potential crosstalk with the Sypro Ruby fluorescence signal, we excluded bands that corresponded to the molecular weight of GS and GP from densitometric measurements (compare white frame–here for the GS signal).
(TIFF)

S3 Fig A and B show the original images for Western blots presented in Figs 1H and 3I, respectively.
(TIFF)
