# Supplementary figures and images for: Metabolic enzymes in glial cells of the honeybee brain and their associations with aging, starvation and food response
Source: PLoS One. 2018 Jun 21;13(6):e0198322. doi: 10.1371/journal.pone.0198322 (PMC6013123; doi:10.1371/journal.pone.0198322)

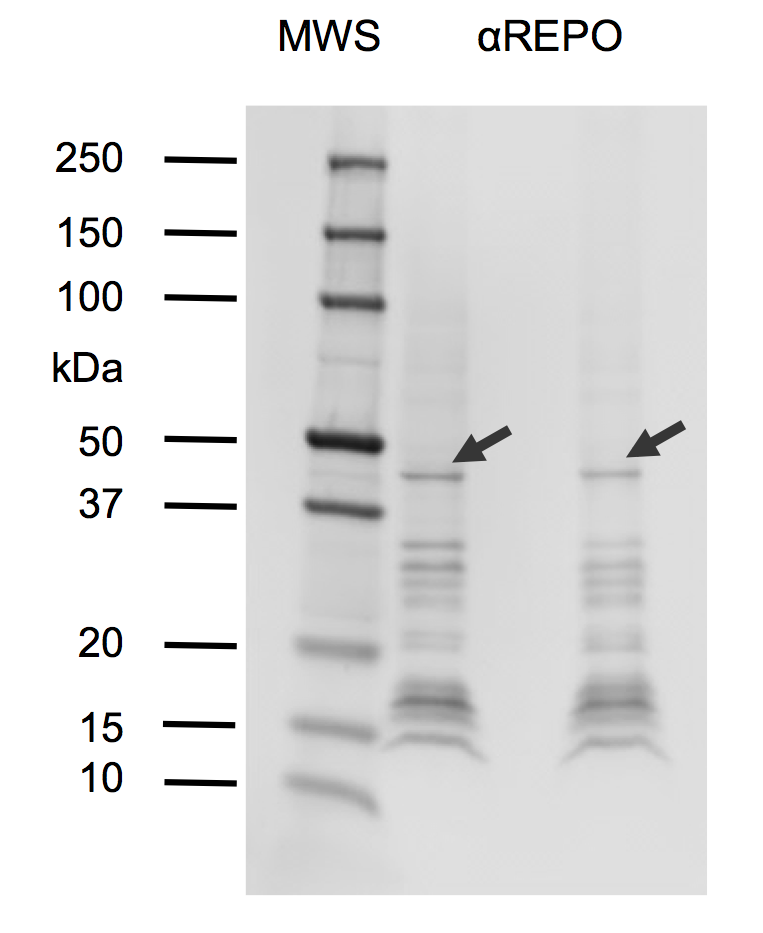

Supplement: S1 Fig — The α-repo serum was previously used for anatomic localization of glial cells in different insect species, including the honey bee, and the resulting anatomic data has been shown to conform with alternative glial localization approaches ([11,52,53,84,85]; for an overview on repo as a glial marker target see [86]). To our knowledge, however, no data is published that demonstrates the specificity of the antibody serum with Western blotting. We found that Western blotting of nuclear extracts from nurse brains revealed multiple bands, all below 50kDa with the largest size band at ca. 45 kDa (arrows, 2 representative blots for N = 10 brain samples). To predict the size of the putative honey bee protein, we performed a BLAST against the Drosophila sequence (NCBI BLAST, http://blast.ncbi.nlm.nih.gov/Blast.cgi, RRID:SCR_004870) and found the result to be consistent with the recently annotated gene retinal homeobox protein Rx2/repo; (LOC410151, A. mellifera). With 48.7kDa, the calculated molecular weight of the predicted protein product (A. mellifera retinal homeobox protein Rx2, XP_016772105) is considerably lower than in Drosophila (70kDa, [53]) but approximates the size of the largest size band that we have detected in Western blots (arrows). However, our Western blots also reveal a number of additional bands below 30kDa, with a prominent band at ca. 18kDa. While we cannot rule out that these may indicate a relatively low specificity of the antibody, we report that all additional bands have a lower molecular weight than the largest size band at ca. 45kDa. This suggests that these bands represent degradation products of the full-size protein. Such degradation may be due to the more extensive lysate treatment that was needed to collect nuclear fractions of brain extracts (below) and, hence, to account for the nucleus-specific localization of the repo protein (compare [53]). Nuclear fractionation. To collect nuclear extracts, we essentially used a hypotonic buffer based prot [file pone.0198322.s001.tiff]

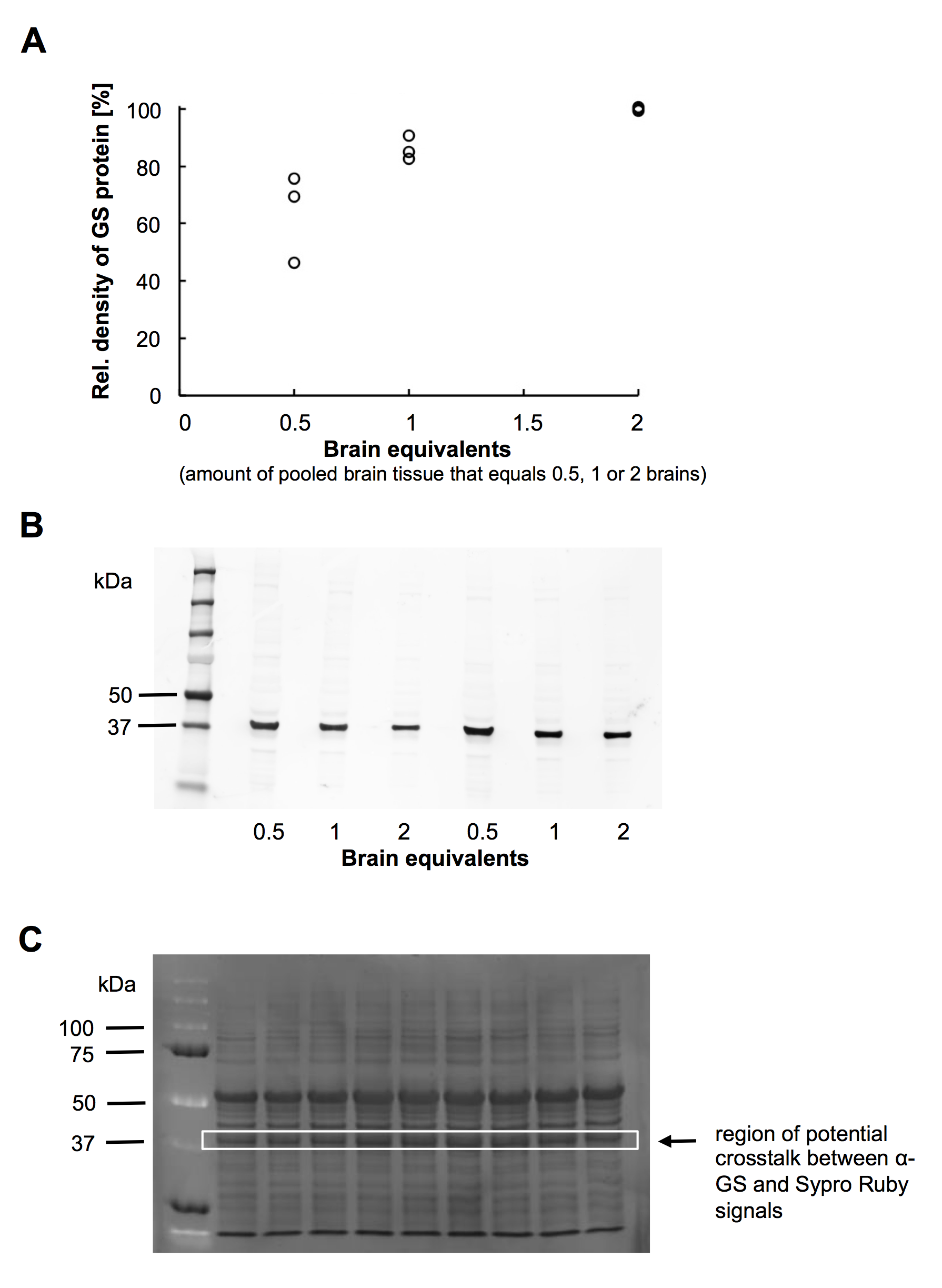

Supplement: S2 Fig — (A, B) Titration tests with antibodies confirm that protein extracts that are equivalent to 0.5 and 1 brain do not saturate the densitometric measurement system (shown here for the α-glutamine synthetase, α-GS, antibody). Prior to semi-quantitative Western blot quantification (compare Figs 4–6) with the two antibodies used, we tested their densitometric response in relation to the amount of brain tissue (‘brain equivalents’) used for gel loading. To this end, 3 independent samples, each with two brains, were pooled (‘stock’) and were then diluted with Laemmli buffer. The resulting protein samples were equivalent to the protein amount of 2 brains (‘stock’), of 1 brain and of ½ brain. (A) Relative protein abundance of GS (in %) shown for three dilutions series, each normalized to the densitometric values measured for the protein ‘stock’ sample, i.e., for the 2-brain equivalent. We found that densitometric values for 1 and 1/2 brain equivalents are smaller than for the 2 brain equivalents. Hence, tissue amounts used to compare different experimental groups (Figs 4–6) will not cause unwanted saturation effects. (B) Representative Western blot showing titration series labeled with the α-GS antibody, and reveal that the GS-band at ca. 41kDa correlates with protein sample concentration. (C) As a reference for normalizing α-GS and α-GP signals to the total fraction of soluble proteins loaded, we re-labeled all blots with Sypro Ruby Protein Blot Stain (Life Technologies Corp.). To avoid potential crosstalk with the Sypro Ruby fluorescence signal, we excluded bands that corresponded to the molecular weight of GS and GP from densitometric measurements (compare white frame–here for the GS signal). (TIFF) [file pone.0198322.s002.tiff]

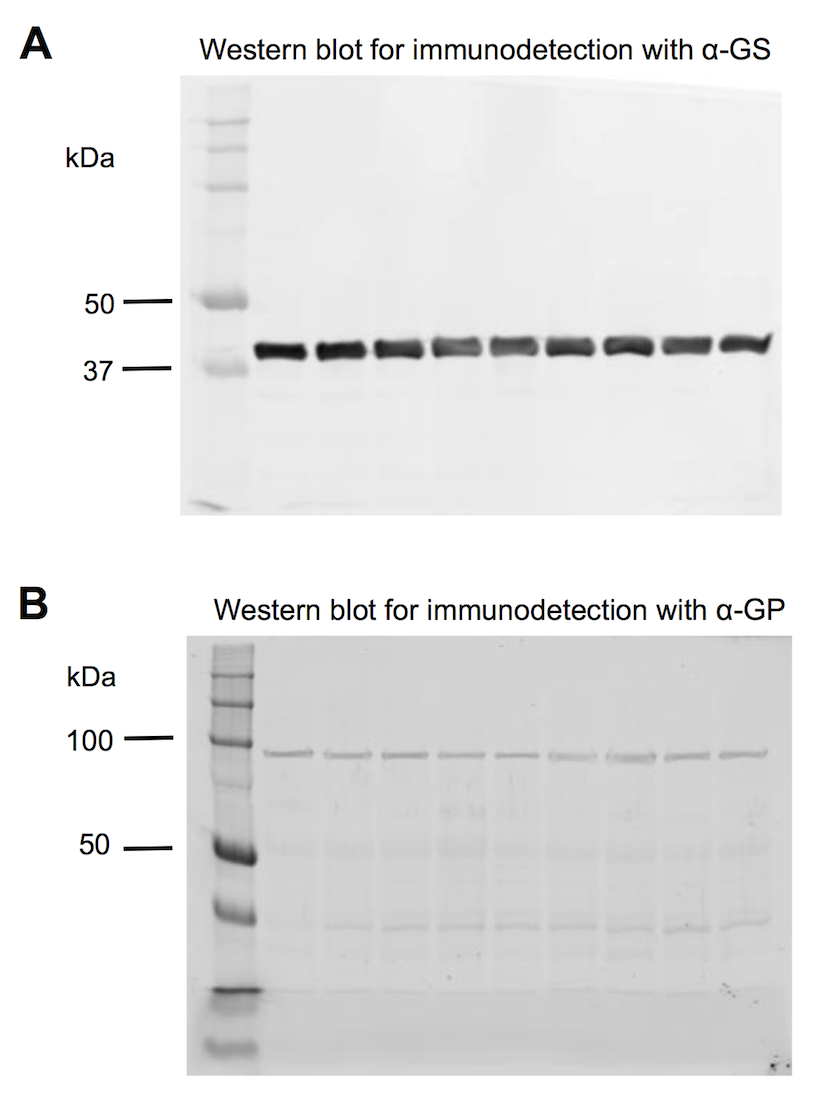

Supplement: S3 Fig — A and B show the original images for Western blots presented in Figs 1H and 3I, respectively. (TIFF) [file pone.0198322.s003.tiff]
